# Positivity of repeat nasal MRSA PCR screening: a single-center experience

**DOI:** 10.1017/ash.2023.491

**Published:** 2023-12-04

**Authors:** Ali Earl, Sage Greenlee, Karen Fong, Hannah Imlay, Emily S. Spivak

**Affiliations:** 1 Department of Pharmacy, University of Utah Health, Salt Lake City, UT, USA; 2 Division of Infectious Diseases, Department of Internal Medicine, University of Utah School of Medicine, Salt Lake City, UT, USA

## Abstract

Repeating nasal methicillin-resistant *Staphylococcus aureus* (MRSA) polymerase chain reactions (PCRs) within 14 days may increase healthcare costs and inform anti-MRSA antibiotic therapy without known benefit. Within an inpatient admission, our retrospective, single-center evaluation found that conversion from negative to positive on repeat nasal MRSA PCR screen was uncommon (2%).

## Introduction

Society guidelines recommend empiric antibiotic coverage of methicillin-resistant *Staphylococcus aureus* (MRSA) when treating hospital and community-acquired pneumonia in areas with high local prevalence of MRSA or presence of risk factors.^1,2^ Nasal molecular screening for MRSA is a useful tool to aid in de-escalation of MRSA-active antibiotic therapy in the treatment of respiratory infections due to its high negative predictive value (NPV) (95–99%) for MRSA pneumonia.^3–7^ The duration of time from specimen collection that a negative screening result can be used to infer MRSA negativity is unclear. Recent data based on concordance of nasal MRSA polymerase chain reaction (PCR) screening with respiratory cultures suggest high predictability up to 14 days from initial collection; however, there is no data on concordance of repeat nasal MRSA PCRs within 14 days.^3^

Despite evidence that nasal MRSA PCR screening retains NPV when compared to culture within 14 days of collection, use of repeat testing is likely. Given potential impacts of repeat testing on healthcare costs, anti-MRSA antibiotic therapy, and implications for antimicrobial stewardship efforts, we set out to describe practice patterns around repeated ordering of nasal MRSA PCRs and the rate of conversion on repeat tests from negative to positive.

## Methods

This single-center, retrospective review included inpatient encounters at an 806-bed academic medical center in Utah. Encounters from March 31, 2021 through March 31, 2023 with the procedure “Methicillin Resistant *Staphylococcus aureus* (MRSA) by PCR, nasal” were included. Additional details, such as ordering service, date and time of collection, and test result were analyzed. All data were collected via the electronic health record.

Patients with only one nasal MRSA PCR during the study period were excluded. Although cross encounter results were included, primary evaluation considered tests for the same patient collected during separate inpatient encounters as a new event. Results were categorized by time from the most recent preceding test result during the same encounter. The first PCR during an encounter equaled time zero and following results were categorized into the predetermined categories of ≤ 24 hours, 25 hours to 72 hours, 73 hours through 7 days, 8 days through 14 days, and exceeding 14 days. Conversion from negative to positive result was determined by comparison of the most recent preceding PCR within the same encounter.

## Results

Over the 2-year period, 5,324 patient encounters included at least one nasal MRSA PCR and 581 encounters (10.9%) included repeat testing. There were 1,342 tests associated with the 581 encounters, of which 761 (56.7%) were repeated during the same admission. The median number of PCRs per encounter was 2 (IQR [2, 2]). The most common medical services ordering repeated tests within 14 days were medical intensive care (17.1%), cardiothoracic surgery (15.5%), and internal medicine (13.6%). Five-hundred and fifty-eight (96.0%) initial nasal MRSA PCRs were negative. Of the 735 PCRs following an initial negative, 183 were completed more than 14 days after the most recent. Most PCRs within 14 days were collected between 7 and 14 days (50.9%). The rate of conversion from negative to positive at any time was 2.0% (15/735). The rate of conversion to positive for those repeated less than 14 days from a negative nasal MRSA PCR was 0.9% (5/552) (Table 1). Only one of five converted PCRs isolated MRSA on respiratory culture. All encounters had respiratory cultures within 96 hours of the repeated test, 60% (3/5) isolated gram-negative organisms with specific verbiage ruling out MRSA, and 100% received vancomycin for any duration with a median of 7 calendar days (IQR [4,7]). The encounter with an MRSA-positive culture had previously isolated MRSA on culture 4 days prior to the repeated test.

**Table 1.** Nasal MRSA PCR screenings associated with conversion from negative to positive

Results converted from negative to positive, *n* (%)	Time from most recent nasal MRSA PCR					
	≤24 h	>24 and ≤72 h	>72 h and ≤7 d	>7 d and ≤14 d	≤14 d	>14 d
No	76 (100)	33 (100)	159 (98.1)	279 (99.3)	547 (99.1)	173 (94.5)
Yes	0 (0)	0 (0)	3 (1.9)	2 (0.7)	5 (0.9)	10 (5.5)

MRSA: Methicillin-resistant *Staphylococcus aureus*; PCR: polymerase chain reaction.

## Discussion

Repeat nasal MRSA PCRs detected rare conversions from negative to positive results within 14 days. Although the NPV for ruling out MRSA pneumonia has repeatedly been demonstrated,^3–6^ positive nasal MRSA PCRs have shown low positive predictive value (35–45%) suggesting limited value for diagnosing MRSA pneumonia.^6,7^ The rare conversion from negative to positive within 14 days, low rate of MRSA isolation from culture among converted results, and the poor positive predictive value indicate repeating nasal MRSA PCRs within 14 days is of limited utility. Additionally, these results suggest that escalation to anti-MRSA therapy for pneumonia within 14 days of a negative nasal MRSA PCR is likely not indicated, as also noted by Turner et al.^3^

To our knowledge, no published studies have evaluated nasal MRSA PCR concordance to previously collected molecular results. Turner et al. evaluated the impact of timing on the predictive value of nasal MRSA PCR in relation to positive respiratory cultures. A high NPV of nasal MRSA PCR overall (94.9%) was reported with consistent results across all cohorts (within 24 hours, 25 to 48 hours, 49 hours to 7 days, 8 to 14 days, and more than 14 days). No difference in predictive value based on level of care or culture specimen was identified.^3^ Palisano et al. compared patients with concordant nasal MRSA screening to respiratory cultures with those found to have discordant (negative nasal MRSA PCR and a positive MRSA respiratory culture) results to identify risk factors for discordance. A history of MRSA infection and greater than 7 days between nasal screen and respiratory culture collection were associated with discordant results; however, high-quality respiratory samples should be pursued in clinically suspected MRSA pneumonia rather than repeated nasal MRSA PCR due to continued high NPV of nasal screens beyond 7 days.^5^ These results support our findings by describing an overall high NPV (94.9%) within 14 days and low rate of discordance (0.28%) indicating that repeating nasal screening without respiratory culture should not alter treatment.^3,5^

This evaluation has several limitations. The dates included periods with high incidence of patients infected with Delta and Omicron severe acute respiratory syndrome coronavirus 2 (SARS-CoV-2) variants. The surge of infections with these variants may have resulted in fewer nasal MRSA PCRs being collected due to admission for known viral illness, thus underestimating rates of repeat testing and/or MRSA. Additionally, infection precautions implemented during the pandemic may have influenced PCR conversion. Chart review was not performed which limited our ability to include respiratory microbiological data for all results; however, literature has already explored predictive values associated with MRSA PCRs and respiratory cultures as noted throughout. Medication administration data for all encounters was not collected, so we were unable to identify whether MRSA PCR was repeated to discourage empiric MRSA-active antibiotic use. Nevertheless, our data suggest that re-initiation of anti-MRSA therapy is likely unwarranted within 14 days of a negative nasal MRSA PCR given low rates of conversion to positive and four of five converted results without isolation of MRSA from culture. Finally, the small incidence of negative to positive conversions precluded the assessment of factors associated with conversion. However, it is unclear if additional data would further affect findings, given the persistently high NPV demonstrated until and potentially beyond 14 days from initial screen.

In summary, these data suggest that nasal MRSA PCRs should not be repeated within 14 days of a prior test. Local antimicrobial stewardship interventions should be implemented to limit repeated nasal MRSA PCRs and empiric MRSA-active antibiotics within 14 days of a negative screen, potentially through the use of clinical decision support tools.
